# Association of phase angle with sarcopenia and muscle function in patients with COPD: a case-control study

**DOI:** 10.1186/s12890-023-02814-9

**Published:** 2024-01-06

**Authors:** Neda Valisoltani, Hamed Mohammadi, Rasoul Aliannejad, Fatemeh Naeini, Asma Rajabi Harsini, Erfan Sadeghi, Pouya Mirzaee, Hossein Imani

**Affiliations:** 1 Department of Clinical Nutrition, School of Nutritional Sciences and Dietetics, Tehran University of Medical Science, Tehran, Iran; 2 Department of Pulmonary and Critical Care, School of Medicine, Shariati Hospital, Tehran, Iran; 3 Research Consultation Center (RCC), Shiraz University of Medical Sciences, Shiraz, Iran; 4 Department of Medicine, Faculty of Medicine, Semnan University of Medical Sciences, Semnan, Iran

**Keywords:** Phase angle, Sarcopenia, Muscle function, Chronic obstructive pulmonary disease

## Abstract

**Background and aims:**

The predictive value of phase angle for sarcopenia diagnosis has been discussed for years. The present investigation was conducted to determine the association between phase angle and sarcopenia in patients with COPD.

**Methods:**

In this case-control study, 222 smoker men were divided into healthy and COPD groups. COPD was diagnosed by a pulmonologist through spirometry. Anthropometric indices, phase angle, muscle function, sarcopenia, and dietary intake were assessed.

**Results:**

A significant inverse association was observed between phase angle and sarcopenia after adjustment for age and energy intake (OR: 0.31, 95% CI 0.18–0.52) and after adjustment for BMI (OR: 0.31, 95% CI 0.18–0.52). A significant decrease was detected in anthropometric indices and indicators of sarcopenia and muscle function in COPD cases compared to the healthy controls.

**Conclusions:**

Although further studies are suggested, phase angle might be considered an indicator of sarcopenia and muscle function in COPD patients.

## Introduction

Chronic obstructive pulmonary disease (COPD) is an increasing public health concern which is characterized by progressive airflow restriction and alveoli inflammation that could be irreversible [1, 2]. The worldwide burden of COPD has been trending upward in the last decades [3]. Due to increased exposure to COPD risk factors and population development, as claimed by the World Health Organization (WHO), COPD is ranked as third cause of mortality in 2030 [4]. In Iran, COPD has the total incidence estimation of 3-6.7% which is close to global average of 2.8–13.9% [5]. Many causative factors are recognized for COPD such as long-term exposure to dusts, indoor and outdoor air pollution and chemicals, but obviously smoking is the most significant risk factor in the onset of COPD [6]. Tobacco smoking leads to reactive oxygen species (ROS) production which can stimulate inflammatory processes [7]. The known symptoms of COPD are cough, excessive sputum, dyspnea and other respiratory diseases such as pneumonia [8]. Due to inflammatory nature of COPD, weight loss, muscle atrophy, and tissue depletion followed by muscle dysfunction and sarcopenia are not farfetched [9]. Timely diagnosis is valuable to prevent the progression of these complications which imposed high costs on patients and society [10].

Many methods are available for the assessment of sarcopenia such as tomography, MRI and dual energy x-ray absorptiometry. Although, these methods possess several imperfections and they aren’t clinically useful [11]. Over the last few years, phase angle (PhA), one of the bioelectrical impedance analyses (BIA) obtained parameters, has been used as a novel prognostic factor for muscle mass reduction and impaired muscle function named sarcopenia. Contrary to old procedures, it is simple, non-invasive and portable. Phase angle by manifesting cell mass and membrane integrity, can accurately define skeletal muscular tissue and muscle strength [12–14]. Numerous research projects were conducted to explore the relationship between phase angle, muscular function and sarcopenia in different disorders. Although many previous studies have indicated independent impact of phase angle on sarcopenia [11, 15–17], there are some controversial findings in this regard. In a cross sectional study on physically active older women, phase angle indicates no relation with sarcopenia [18]. Moreover, another cross-sectional study on elderly hospitalized patients, introduced phase angle as an imprecise marker for predicting muscle mass, muscular power, functional status and sarcopenia [19].

Due to the conflicting results of prior studies, the importance of utilizing predictive tools for preventing COPD complications specially sarcopenia and muscle weakness, increasing patients’ quality of life, and decreasing social costs, the present study was performed to examine the association of phase angle with sarcopenia in COPD patients and healthy men who were referred to Shariati hospital, Tehran, Iran. As mentioned, although there were several cross-sectional studies in this regard, this is the first research with hospital-based case-control design that selected 111 healthy smokers as controls. Up to now, there was no study with case-control design that assessed the association of phase angle with sarcopenia.

## Methods

### Study design and population

In this hospital-based case control study, men aged 40–70 years with a history of smoking were recruited between September 2021 and October 2022 at Shariati hospital, Tehran, Iran. Both cases and controls were selected by random sampling method. The case group was included 111 COPD patients who were confirmed by a pulmonologist using spirometry report (FEV1/FVC < 0.7). We randomly selected 111 healthy smokers in the same location for the control group. Convenience method was used for sampling. The case and control groups were matched by age and body mass index (BMI).

The case group entrance criteria were as: (1) willingness to cooperate, (2) age between 40 and 70 (3) COPD diagnosis according to spirometry test (4) smoking habit. Our exclusion criteria were included: (1) recurrence of disease in the last 4 weeks, (2) suffering from underlying chronic diseases such as hepatic cirrhosis, kidney failure, heart failure and coronary heart disease, unchecked thyroid abnormalities, autoimmune disorders such as rheumatoid arthritis, cancer within the past 3 years, various infections (hepatitis, HIV, etc.) and any lung diseases except COPD (The rational for excluding these subjects is that the mentioned chronic disorders due to their inflammatory and degenerative nature could significantly affect assessed outcomes including muscle function [20].) (3) long-time steroids medicine taking. The control group met the same criteria, except for the omission of COPD diagnosis. All participants were informed about the purpose of the study and the protocol at the beginning. Study methods were carried out in accordance with relevant guidelines and regulations. We collected written signed informed consent forms from all subjects. All experimental protocols were approved by Tehran University of Medical science (TUMS) ethics committee (number: IR.TUMS.MEDICINE.REC.1400.828).

The required sample size in this case-control study was calculated using the effect size of previous studies (equal to 0.37) and considering the significance level of 5% and the power of 80% in G*Power software. Therefore, the minimum sample size required to conduct this research was estimated as 222 subjects in total (111 members in each group).

### Assessment of demographic and general information

Prior to study commencement, a general questionnaire was applied to collect the participants’ information regarding age, marriage status (single/married), educational level (less than diploma/higher or equal to diploma), occupational status (employee, laborer, self-employed), residency (village/industrial city), smoking status (non-smoker/ex-smoker/current smoker), smoking history of family members, hookah using, duration of COPD (years) and history of underlying disorders (kidney, heart, liver disease as well as neoplasm malignancies, edema and ascites).

### Pulmonary function assessment

First, all individuals were directed to laboratory which was located in pulmonary ward in Shariati hospital, Tehran, Iran for performing spirometry test. After signing the written consent form, completing registration and general questionnaire, a trained technician explained the accurate procedure of the test. The test was repeated until the subjects did it correctly. Finally, FEV1 and FEV1/FVC were obtained from the report for COPD diagnosis. According to GOLD 2018 pocket guide, COPD was diagnosed based on FEV1/FVC < 0.7 and airflow limitation severity was classified base on post-bronchodilator FEV1 in patients with FEV1/FVC < 0.7 (mild: FEV1 ≥ 80%, moderate: 50% ≤ FEV1 < 80%, severe: 30% ≤ FEV1 < 50%, very severe: FEV1 < 30%) [21, 22].

### Anthropometric assessment

Weight was measured in fasting circumstance, bare feet, with minimal clothing and using a digital scale (Seca, Hamburg, Germany) with an accuracy of 100 g. The height was evaluated in standing position, without shoes to the nearest of 0.5 cm by a measuring tape which was installed on the wall. Body mass index (BMI) was calculated by using the formula of dividing weight in kilograms by the height in square meters (weight/height^2^ (kg/m^2^)).

### Bioelectrical impedance analysis and phase angle measurement

Body composition was measured by bioelectrical impedance analysis device (InBody 770, Korea), which is a simple and non-invasive technique. All participants were refrained from eating, drinking, physical activity and vigorous exercise before performing the test. Due to the use of 8 electrodes (two under the right and left leg and two for the right and left hand), this device provides the possibility of separate analysis for different organs (right and left hands and right and left legs) and trunk. Skeletal muscle mass (SMM), body fat mass (BFM), total body water (TBW), soft lean mass (SLM) and fat free mass (FFM) were obtained from bioelectrical impedance result paper.

Phase angle was calculated by the device using resistance (R) and reactance (Xc) at 50 kHz by the following equation [23]:

$$PhA \left(degree\right)= \text{a}\text{r}\text{c}\text{t}\text{a}\text{n}\text{g}\text{e}\text{n}\text{t} (\text{R}/\text{X}\text{c}) \times \left(\frac{180}{\pi }\right)$$

### Muscle function assessment

Hand grip strength, 5STS and 6MWD were measured to assess muscle function. For the five times sit-to-stand test (5STS), subjects sat on a specific chair with the folded arms across their chest and the hands touching the shoulders with a straight back position. Then, we asked them to stand up and sit down for 5 times continuously, in the earliest possible time, without any help. we take the time in seconds by a mobile phone timer. The fastest time was recorded after twice repeating test [24]. Moreover, an isometric hand grip dynamo-meter was utilized to measure hand grip strength. All participants performed three trials in a sitting position, while their folded elbows were placed next to their body and their wrists were in a neutral position. The maximum value among the three trials was reported as a reliable data for each hand [25]. In the six minutes walking distance (6MWD) test, the individuals were asked to walk as fast as they can without running for 6 min in 30 m fixed flat unobstructed corridor. They were given the opportunity to slow down or stop to rest if it was necessary. SpO2 was measured at starting and finishing point by a portable pulse oximeter device [26].

### Sarcopenia assessment

According to the revised European consensus on definition and diagnosis of sarcopenia, subjects were classified as sarcopenic if they have two factors: low muscle mass and low muscle strength. Initially, muscle strength was assessed by an isometric hand grip dynamo-meter as noticed. Mean value of two hands was a representative of whole body hand grip strength. If it was less than 30 kg, it categorized as weak muscle strength. In the next step, muscle mass was computed by bioelectrical impedance device as above explanation. skeletal muscle index (SMI) was acquired by using the following formula: SMI = SMM/height^2^. SMI under 10.8 kg/m^2^ was considered as low muscle mass. Finally, participants who fulfil both criteria were reported as sarcopenic for further analysis [27].

### Dietary assessment

A valid and reliable 168 items food frequency questionnaire (FFQ), the most cost-effective, time-saving, and practical tool for nutritional epidemiology studies, was applied to assess the participants’ dietary intake [28, 29]. An expert dietitian interviewed face to face with all men. They were asked to report the average frequency of each food consumption base on daily, weekly, monthly or yearly schedule through the past year. All records were changed to grams per day by household program of Iranian foods. The modified nutritionist 4 software was applied to elicit the nutrient contents of foods.

### Statistical analysis

Statistical data were analyzed using SPSS version 24 software and the results were reported as mean ± standard deviation (SD) or number (percentage). The normality of the distribution of the variables were checked by the Kolmogorov-Smirnov test. If the distribution of the variables were normal, Independent sample t-test test was run to compare the average between test groups. Also, Mann-Whitney test was used for variables without normal distribution. Also, to compare qualitative variables between groups, Chi-Square test was run. P values less than 0.05 was regarded statistically significant.

## Results

Mean age of the COPD cases and controls were 56.09 and 53.87, respectively. As demonstrated in Table 1, general characteristics of study participants including duration of the disease, physical activity, and the values of FEV1 and FEV1/FVC were significantly different between the study groups (P < 0.001). Patients with COPD possessed lower FEV1, FEV1/FVC, and physical activity values in comparison with healthy controls.

**Table 1:** General characteristics of COPD cases and controls

Variables	COPD (n = 111)	Control (n = 111)	P-value^*^
Age	56.09 ± 8.95	53.87 ± 9.58	
Duration of disease	5.23 ± 3.91	0.00 ± 0.00	< 0.001
MET	2040.91 ± 1590.29	5713.79 ± 2370.42	< 0.001
FEV1	63.16 ± 18.23	88.00 ± 10.61	< 0.001
FEV1/FVC	60.95 ± 10.02	80.94 ± 4.94	< 0.001

Values are indicated as mean ± standard deviation

Independent sample T-test was applied for groups comparison

*BMI* body mass index, *MET* metabolic equivalent, *FEV1* forced expiratory volume in 1 s, *FVC* forced vital capacity

*The p < 0.05 was considered as significant

Table 2 presented the mean and standard deviation of the dietary intakes of study participants. There was significant difference between the case and control groups in terms of energy, protein, fat, dietary fiber, water-soluble vitamins, zinc, and iron intake (P < 0.05). A significant decrease was found in the consumption of energy, protein, fat, dietary fiber, water-soluble vitamins, zinc, and iron in patients with COPD compared to the control group. However, vitamin D was proposed to consume more in COPD patients in comparison with healthy men (1.20 ± 0.85 µg vs. 1.13 ± 0.86 µg).

**Table 2:** Dietary status of COPD cases and controls

Variables	COPD (n = 111)	Control (n = 111)	P-value^*^
Energy (Kcal)	1758.57 ± 493.32	1899.00 ± 417.85	0.023
Carbohydrate (Gm)	250.65 ± 74.01	268.20 ± 62.93	0.040
Protein (Gm)	69.35 ± 19.40	76.16 ± 17.25	0.006
Fat (Gm)	56.01 ± 15.20	61.90 ± 13.34	0.002
Total dietary fiber (Gm)	11.19 ± 3.59	12.72 ± 2.92	0.001
Thiamin (mg)	1.70 ± 0.52	1.83 ± 0.43	0.046
Riboflavin (mg)	1.80 ± 0.55	1.99 ± 0.51	0.009
Niacin (mg)	18.08 ± 5.62	20.12 ± 5.02	0.005
Vitamin B6 (mg)	1.07 ± 0.33	1.22 ± 0.32	< 0.001
Folate (Ug)	250.00 ± 75.33	275.48 ± 75.11	0.012
Vitamin D (Ug)	1.20 ± 0.85	1.13 ± 0.86	
Vitamin E (mg)	3.15 ± 1.18	3.44 ± 1.07	0.036
Vitamin A (Ug)	1388.44 ± 769.66	1596.05 ± 763.34	0 0.045
Vitamin C (mg)	67.92 ± 25.97	83.43 ± 27.28	< 0.001
Vitamin B12 (Ug)	7.24 ± 4.70	9.62 ± 5.84	0.001
Zn (mg)	8.15 ± 2.31	8.89 ± 1.96	0 0.010
Ca (mg)	763.89 ± 267.72	788.21 ± 221.21	
Fe (mg)	13.19 ± 4.05	14.33 ± 3.43	0.024

Values are indicated as mean ± standard deviation

Independent sample T-test was applied for groups comparison

*The p < 0.05 was considered as significant

As shown in Table 3, anthropometric indices including weight, height, SLM, FFM, SMM, TBW, and phase angle were significantly different between the study groups (P < 0.02). All the mentioned variables were significantly lower in COPD cases compared to the healthy subjects. Also, BMI was indicated to be lower in patients with COPD compared to the controls (24.89 ± 5.02 kg/m^2^ vs. 25.90 ± 3.79 kg/m^2^). However, an increase was detected in PBF and BFM in COPD cases in comparison with healthy men (24.98 ± 9.50% vs. 23.41 ± 7.00% and 24.98 ± 9.50 kg vs. 23.41 ± 7.00 kg, respectively).

**Table 3:** Anthropometric status of COPD cases and controls

Variables	COPD (n = 111)	Control (n = 111)	P-value^*^
Weight (kg)	73.52 ± 15.94	78.66 ± 13.79	0.011
Height (cm)	171.76 ± 7.53	174.06 ± 6.94	0.018
BMI (kg/m^2^)	24.89 ± 5.02	25.90 ± 3.79	
SLM (kg)	51.04 ± 8.00	56.42 ± 8.28	< 0.001
FFM (kg)	54.05 ± 8.45	59.72 ± 8.80	< 0.001
SMM (kg)	29.31 ± 4.98	33.40 ± 5.37	< 0.001
PBF (%)	24.98 ± 9.50	23.41 ± 7.00	
BFM (kg)	24.98 ± 9.50	23.41 ± 7.00	
TBW (kg)	39.87 ± 6.25	43.95 ± 6.42	< 0.001
Phase angle (degree)	5.01 ± 0.66	5.80 ± 0.70	< 0.001

Values are indicated as mean ± standard deviation

Independent sample T-test was applied for groups comparison

*BMI* body mass index, *SLM* soft lean mass, *FFM* fat free mass, *SMM* skeletal muscle mass, *PBF* percent of body fat, *BFM* body fat mass, *TBW* total body water

*The p < 0.05 was considered as significant

Table 4 exhibited the mean and standard deviation of the sarcopenia and muscle function indicators. A significant difference was observed in 6MWD, pre O2, post O2, 5STS, right hand grip, left hand grip, and SMI between the case and control groups (P < 0.001). All the mentioned variables except 5STS were significantly lower in COPD patients compared to the control group. The mean of 5STS was 12.85 s and 8.62 s in COPD cases and healthy controls, respectively. As demonstrated in Table 5, a significant difference in the prevalence of sarcopenia was evident between the case and control groups (P < 0.001). Patients with COPD showed a higher prevalence of sarcopenia compared to the control group (45.8% vs. 11.7%).

**Table 4:** Sarcopenia criteria and muscle function status of COPD cases and controls

Variables	COPD (n = 111)	Control (n = 111)	P-value^*^
6MWT (m)	429.95 ± 76.59	565.97 ± 384.85	< 0.001
pre.O2 (%)	92.66 ± 3.92	96.29 ± 1.17	< 0.001
post.O2 (%)	89.95 ± 5.83	95.50 ± 1.39	< 0.001
5STS (s)	12.85 ± 4.13	8.62 ± 2.13	< 0.001
Right hand.grip (kg)	29.67 ± 7.95	37.81 ± 8.19	< 0.001
Left hand.grip (kg)	28.30 ± 7.86	36.85 ± 8.33	< 0.001
SMI (kg/m^2^)	9.89 ± 1.28	10.97 ± 1.21	< 0.001

Values are indicated as mean ± standard deviation

Independent sample T-test was applied for groups comparison

*6MWT* 6 min walking test, *5STS* 5 sit to stand, *SMI* skeletal muscle index

*The p < 0.05 was considered as significant

**Table 5:** Prevalence of sarcopenia in cases and controls

		Group				P-value
		Control		COPD		
Sarcopenia	No	98	88.3%	58	54.2%	< 0.001
	Yes	13	11.7%	49	45.8%	

Values are number and percent

Chi-square test was applied for groups comparison

*The p < 0.05 was considered as significant

The association of phase angle with sarcoprnia was demonstrated in Table 6. In the crude model, a significant negative association of phase angle with sarcopenia was found (OR: 0.25, 95% CI 0.16–0.41). Also, in the model 1, there was a significant inverse association between phase angle and sarcopenia after adjusting for age and energy intake of the participants (OR: 0.31, 95% CI 0.18–0.52). In addition, after adjusting for BMI of the participants, a significant negative association of phase angle with sarcopenia was observed in the model 2 (OR: 0.31, 95% CI 0.18–0.52).

**Table 6:** Association of phase angle with sarcopenia

Outcome	Predictors	OR (95% CI)^*^
Sarcopenia	Crude	0.25 (0.16–0.41)
	Model 1	0.31 (0.18–0.52)
	Model 2	0.31 (0.18–0.52)

Crude: phase angle as the main predictor

Model 1: further adjusted for age and energy intake

Model 2: further adjusted for BMI

*Logistic regression analysis was used

## Discussion

To our knowledge, the present case-control study examined the association of phase angle as an indicator of malnutrition with sarcopenia in COPD patients and healthy men for the first time. Based on the obtained findings, there was a significant inverse association of phase angle with sarcopenia even after adjusting for confounders, including the participants’ age, energy intake, and BMI. Anthropometric indices and indicators of sarcopenia and muscle function significantly differed between the study groups. A significant decrease was detected in anthropometric indices, including phase angle and sarcopenia and muscle function indicators in COPD cases compared to the healthy controls. Although, in comparison with the control group, there was a significant increase in 5STS in patients with COPD.

In line with our findings, previous studies uncovered the association between phase angle with muscular strength and functional exercise [30]. A recent systematic review by Vincenzo et al. [31] concluded that phase angel is decreased in sarcopenic subjects. Also, they declared that the prevalence of sarcopenia is higher in subjects with low phase angel [31]. A study of 263 COPD patients revealed a significant decrease in phase angle in sarcopenic patients compared to healthy ones [32]. In addition, an investigation on hospitalized geriatric patients finds phase angle as a determinant and a useful diagnostic tool for sarcopenia [11]. Also, based on the main findings of a prospective study, the bioimpedance phase angel predicts low muscle strength, impaired quality of life, and increased mortality in patients with cancer [33]. Another prospective observational study on 250 maintenance hemodialysis patients presented phase angle as an impaired muscle function predictor [34]. Moreover, based on a cross-sectional study on unselected patients with cancer, phase angel is associated with the risk of sarcopenia [35]. Phase angle also emerged as an independent and valuable indicator of muscle strength in COPD patients [36].

Nevertheless, a cross-sectional study of 129 kidney transplantation individuals indicated controversial results. Although phase angle has a positive connection with hand grip strength and functional capacity, it doesn’t relate to sarcopenia and its other indices. In this study, no observed relation between phase angle and sarcopenia components may be under the influence of their design and unmatched variables with participants’ age and BMI, which might restrict the explanation of the results and cause misinterpretation. Also, the discrepancies may stem from differences in measurement tools for phase angle and variations in diagnostic criteria of sarcopenia [37].

The relationship of phase angle with sarcopenia and muscle function has been debated for years. Several preceding studies confirmed our findings based on knowing phase angle as a predictor of sarcopenia and impaired muscle function [32, 38, 39]. In opposition to our final results, a cross-sectional study of COPD patients disclaimed phase angle as an indicator of sarcopenia [40]. Also, Pessoa et al. indicated no association between low phase angle with sarcopenia, low muscle mass, low hand grip strength, and low walk speed [41]. Contrasting noticed results occurred following using dissimilar devices to measure phase angle, joining participants with various age ranges, utilizing different criteria for sarcopenia diagnosis, and performing functional tests by different persons. The association between low phase angle with reduced muscular strength and limited functional exercise, which is generally defined as sarcopenia, was demonstrated in prior studies [30]. There are many feasible mechanisms for its explanation. As phase angle refers to the quantity of cellular mass and the integrity of the cell membrane, a reduction in phase angle levels reveals smaller mascularity and lower water content. For this reason, low handgrip strength, impaired muscle function, less functional capacity, and relatively more sarcopenia expectance occurred as a result of low phase angle [42–44].

### Strengths and limitations

The main strength of our study is the case-control design which allows us to demonstrate the difference in phase angle, sarcopenia indices, and functional status in healthy and COPD men for the first time. Also, the present study is well-characterized with covariate data to perform variable-adjusted analysis. To interpret obtained conclusion, several limitations should be regarded. The small sample size and the non-causal nature of this investigation are among the major limitations. Furthermore, there are dissimilarities in disease severity and duration, socioeconomic status, treatment duration, and years of smoking in the study population. Moreover, the outcome could not be extended to women and younger adults because our participants were men, and all were older than 40. In addition, not using supine bioelectrical impedance analysis device could be considered as another limitation because it seriously affects the reference values. Finally, normal FEV1/FVC ratio can decrease with age and it might affect the results.

## Conclusion

Generally, our results indicated a significant inverse association between phase angle and sarcopenia in COPD cases and healthy subjects. Therefore, it would be concluded that phase angel as an indicator of malnutrition might be considered as a utilizing predictive tools in clinical setting for preventing COPD complications to increase patients’ quality of life and decrease financial burden on health care system. Further robust and well-designed investigations with larger sample sizes are proposed to examine the association of phase angle with sarcopenia and muscle function indicators in COPD patients and healthy subjects. Also, more epidemiological evidence is needed to detect the exact phase angle cut-offs for sarcopenia in different conditions.

## Data Availability

The datasets used and analyzed during the current study is available from the corresponding author on reasonable request.

## List of abbreviations

COPD: Chronic obstructive pulmonary disease
WHO: World Health Organization
ROS: Reactive oxygen species
MRI: Magnetic resonance imaging
PhA: phase angle
BIA: bioelectrical impedance analysis
BMI: body mass index
FFM: fat free mass
SLM: soft Lean mass
SMM: skeletal muscle mass
SMI: skeletal muscle index
BFM: body fat mass
BF%: body fat percentage
5STS: five times sit-to-stand test
6MWD: six minutes walking distance
SpO2: Saturation of peripheral oxygen
FFQ: food frequency questionnaire
N4: Nutritionist IV software
